# Anti-angiogenic drugs: direct anti-cancer agents with mitochondrial mechanisms of action

**DOI:** 10.18632/oncotarget.20858

**Published:** 2017-09-13

**Authors:** Lewis A. Quayle, Maria G. Pereira, Gerjan Scheper, Tammy Wiltshire, Ria E. Peake, Issam Hussain, Carol A. Rea, Timothy E. Bates

**Affiliations:** ^1^ School of Life Sciences, Joseph Banks Laboratories, University of Lincoln, Lincoln, LN6 7DL, U.K.; ^2^ Department of Oncology and Metabolism, Medical School, University of Sheffield, Sheffield, S10 2RX, U.K.; ^3^ School of Pharmacy, Joseph Banks Laboratories, University of Lincoln, Lincoln, LN6 7DL, U.K.; ^4^ Drugs With A Difference Limited, BioCity Nottingham, Nottingham, NG1 1GF, U.K.; ^5^ Marlin Therapeutics Limited, Nottingham Science Park, Nottingham, NG7 2RF, U.K.

**Keywords:** angiogenesis, chemotherapy, breast cancer, lung cancer, mitochondria

## Abstract

Components of the mitochondrial electron transport chain have recently gained much interest as potential therapeutic targets. Since mitochondria are essential for the supply of energy that is required for both angiogenic and tumourigenic activity, targeting the mitochondria represents a promising potential therapeutic approach for treating cancer. Here we investigate the established anti-angiogenesis drugs combretastatin A4, thalidomide, OGT 2115 and tranilast that we hypothesise are able to exert a direct anti-cancer effect in the absence of vasculature by targeting the mitochondria. Drug cytotoxicity was measured using the MTT assay. Mitochondrial function was measured in intact isolated mitochondria using polarography, fluorimetry and enzymatic assays to measure mitochondrial oxygen consumption, membrane potential and complex I–IV activities respectively. Combretastatin A4, OGT 2115 and tranilast were both shown to decrease mitochondrial oxygen consumption. OGT 2115 and tranilast decreased mitochondrial membrane potential and reduced complex I activity while combretastatin A4 and thalidomide did not. OGT 2115 inhibited mitochondrial complex II–III activity while combretastatin A4, thalidomide and tranilast did not. Combretastatin A4, thalidomide and OGT 2115 induced bi-phasic concentration-dependent increases and decreases in mitochondrial complex IV activity while tranilast had no evident effect. These data demonstrate that combretastatin A4, thalidomide, OGT 2115 and tranilast are all mitochondrial modulators. OGT 2115 and tranilast are both mitochondrial inhibitors capable of eliciting concentration-dependent reductions in cell viability by decreasing mitochondrial membrane potential and oxygen consumption.

## INTRODUCTION

Angiogenic neovascularisation is central to the pathogenesis and haematogenous dissemination of malignant tumours [1]. The angiogenic sprouting and intussusceptive growth of newly formed blood vessels is critically dependent on the provision of metabolic energy, in the form of adenosine 5′-triphosphate (ATP), by mitochondrial oxidative phosphorylation (OXPHOS) [2]. A plethora of recent evidence has also demonstrated that the cancer cells within tumours are significantly more reliant on mitochondrial oxidative metabolism to meet their metabolic energy demands than has been generally assumed since the introduction of the Warburg hypothesis almost 60 years ago [2–5]. These observations, when taken together, indicate a pivotal role for mitochondria in the pathogenesis of cancer, making them a promising molecular target for anti-cancer therapies.

It has been demonstrated that mitochondria are unexpected targets for a range of drugs. The widely prescribed anti-diabetic agent metformin has been associated with a reduced incidence of cancer amongst type-II diabetic patients treated with metformin [6]. Data suggests that metformin possesses direct anti-cancer activity mediated by effective inhibition of mitochondrial complex I [7]. In addition, a number of other agents have been identified that act at the mitochondria to cause cancer cell death *in vitro*, including tricyclic antidepressants such as chlorimipramine [8]; vanilloids such as capsaicin [9]; and cannabinoids such as Δ9-tetrahydrocannabinol (THC), the psychoactive compound in *Cannabis sativa* [10].

Several small molecule inhibitors of angiogenesis have been shown to possess both anti-angiogenic and direct anti-cancer properties *in vitro* and *in vivo* [11–16]. Due to the heavy reliance of both angiogenesis and tumorigenesis on mitochondrial function, the ability of these agents to independently target both the tumour vasculature and the malignant cell mass implies that each might have at least one mitochondrial target of action.

In this study we measured the *in vitro* cytotoxicity of the anti-angiogenic drugs combretastatin A4, thalidomide, OGT 2115 and tranilast on MCF-7 human breast cancer and NCI-H460 human non-small cell lung cancer cell lines using the MTT assay. We also investigated the potential underlying cell death modalities by assessing cellular morphology under fluorescence microscopy following staining of cytoskeletal F-actin and nuclei, as well as fluorimetric measurement of cellular caspase-3 activity. In addition, we also measured oxygen consumption and membrane potential in intact isolated mitochondria, and the specific enzyme activities of mitochondrial complex I [EC 1.6.5.3], mitochondrial complex II–III [EC 1.8.3.1] and mitochondrial complex IV [EC 1.9.3.1] in the presence of a range of concentrations of each drug.

## RESULTS

### Anti-angiogenic drugs inhibited the proliferation of MCF-7 and NCI-H460 cells

**Figure 1:**
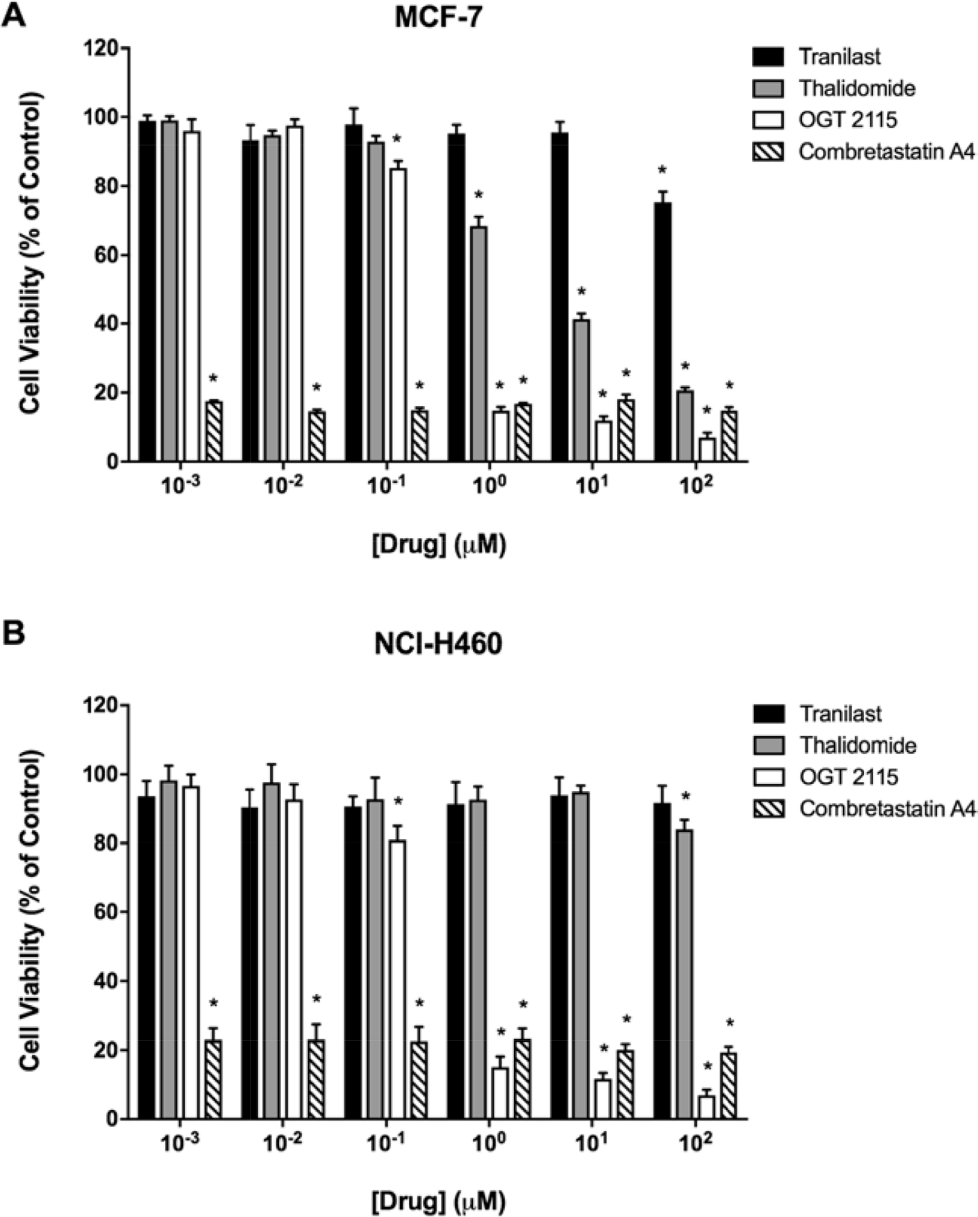
MTT cell viability assays MTT assays demonstrating the relative viability of MCF-7 human breast cancer cells (**A**) and NCI-H460 human non-small cell lung cancer cells (**B**) following a 72-hour period of exposure to a range of concentrations (1 nM–100 μM) of either combretastatin A4 (IC_50_ < 1 nM for MCF-7 and NCI-H460), OGT 2115 (IC_50_ = 0.26 μM for MCF-7 and IC_50_ = 0.24 μM for NCI-H460), thalidomide (IC_50_ = 3.03 μM for MCF-7 and IC_50_ > 100 μM for NCI-H460) or tranilast (IC_50_ > 100 μM for MCF-7 and NCI-H460). Data are expressed as means ± SEM for three independent experiments (*n* = 3). The difference between control and treatment groups at each drug concentration was determined by two-way ANOVA followed by Dunnett’s multiple comparison test. The asterisk symbol (*) is used to denote statistical significance in the difference between experimental and negative control values (*P* ≤ 0.05).

MCF-7 human breast cancer and NCI-H460 human non-small cell lung carcinoma cells were treated with a range of concentrations (1 nM - 100 μM) of each anti-angiogenic drug for 72 hours, after which cell viability was measured by an MTT assay. Figure 1 shows that the viability of both MCF-7 and NCI-H460 cells was reduced at all concentrations of combretastatin A4 used relative to the solvent control (1% DMSO). There was a concentration-dependent decrease in MCF-7 and NCI-H460 cell viability at OGT 2115 concentrations of 0.1 μM and above. When MCF-7 cells were incubated with thalidomide there was a significant concentration-dependent decrease in cell viability at drug concentrations above 1 μM, while NCI-H460 cell viability was only reduced at a thalidomide concentration of 100 μM. Tranilast only caused a significant decrease in viable MCF-7 cell number at a concentration of 100 μM, while no reduction in viable NCI-H460 cell mass was evident at any of the concentrations of tranilast used.

### Fluorescence microscopy showed changes in cytoskeletal and nuclear morphology

**Figure 2:**
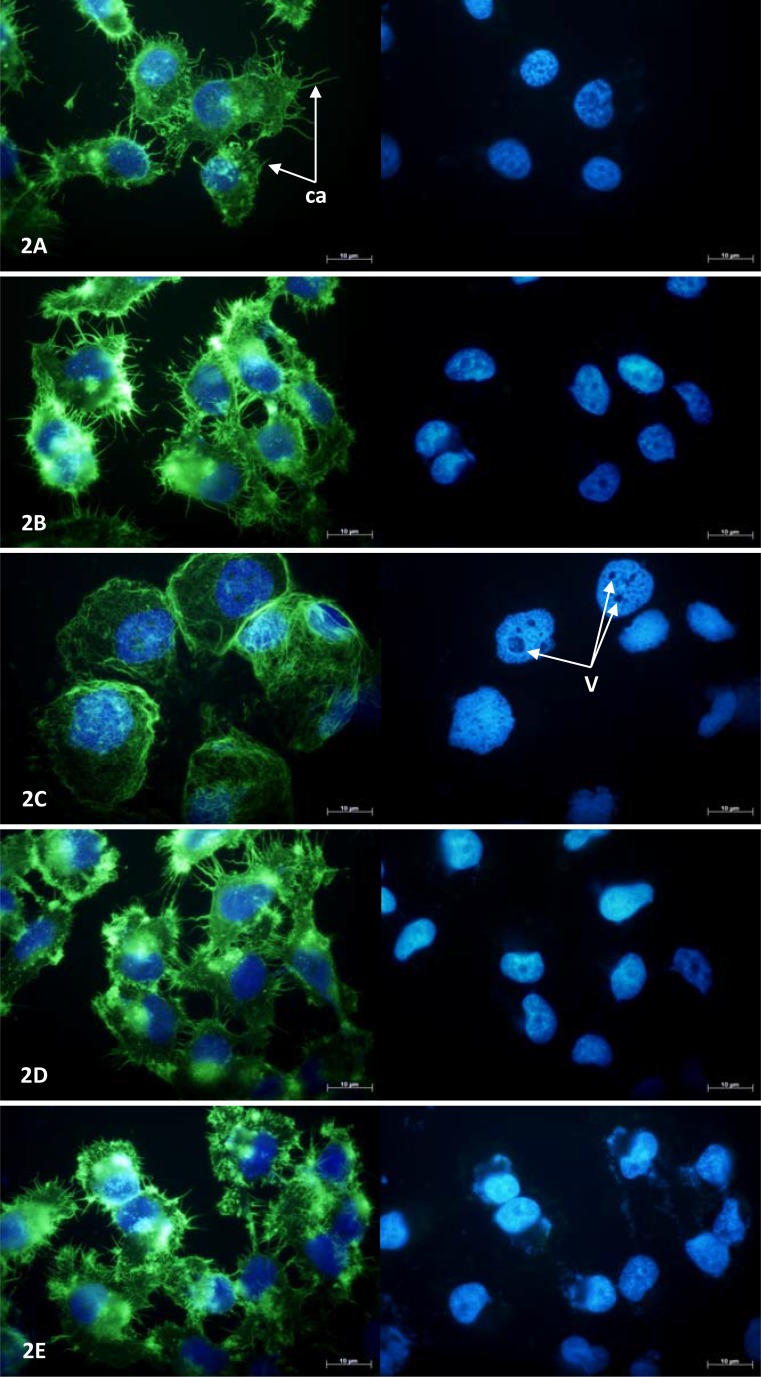
MCF-7 cell morphology following 24-hours exposure to anti-angiogenic drugs Fluorescence photomicrographs illustrating NCI-H460 cell cytoskeletal and nuclear morphology following a period of 24 hours exposure to 100 μM combretastatin A4 (**A**) 100 μM thalidomide (**B**) 100 μM OGT 2115 (**C**) 100 μM tranilast (**D**) and control cells exposed to 1% DMSO (**E**). Cytoskeletal F-actin fibres are stained with AlexaFluor488-phalloidin (green) and superimposed on the nuclear dsDNA stained with DAPI (blue). v = vacuole-like structures, ca = cytoskeletal attachments.

**Figure 3:**
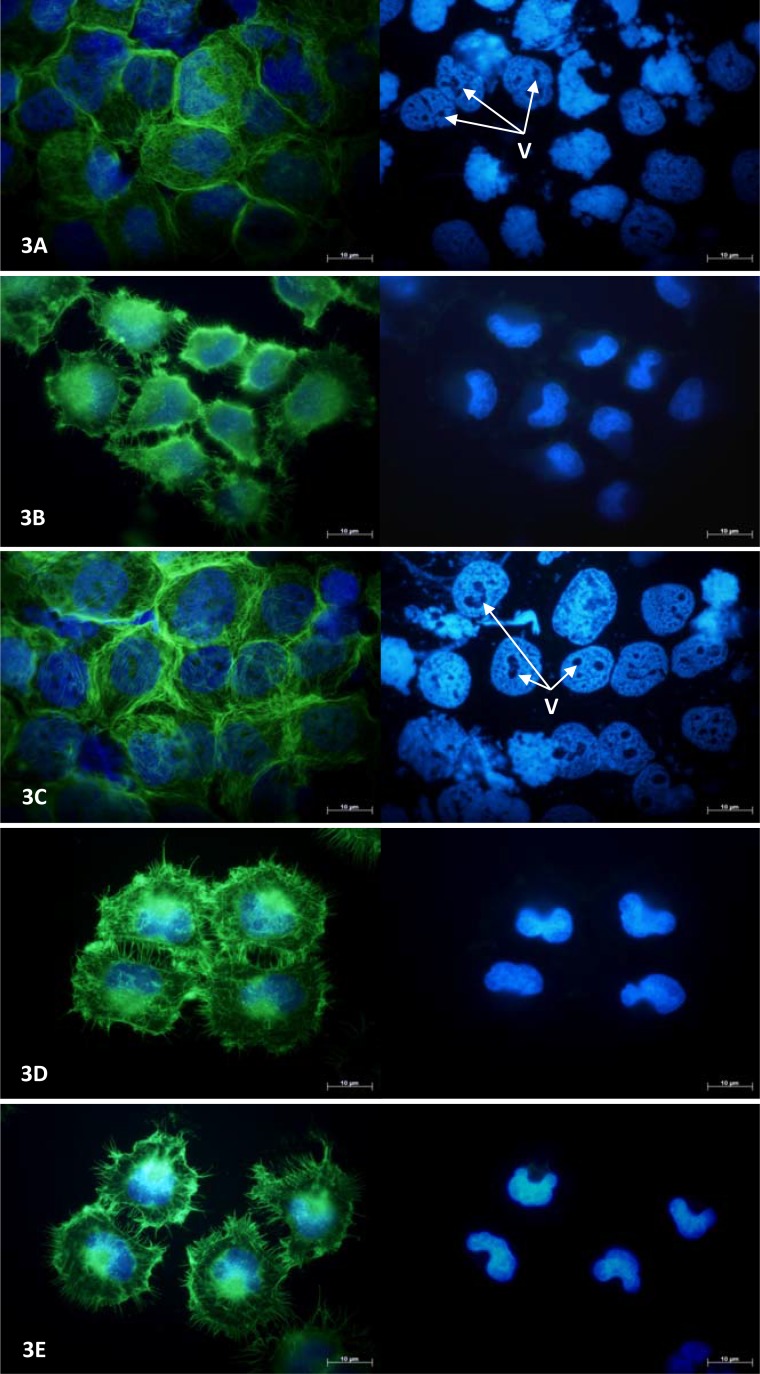
NCI-H460 cell morphology following 24-hours exposure to anti-angiogenic drugs Fluorescence photomicrographs illustrating cytoskeletal and nuclear morphology of MCF-7 cells following a period of 24 hours exposure to 100 μM combretastatin A4 (**A**) 100 μM thalidomide (**B**) 100 μM OGT 2115 (**C**) 100 μM tranilast (**D**) or and control cells exposed to 1% DMSO (**E**). Cytoskeletal F-actin fibres are stained with AlexaFluor488-phalloidin (green) and superimposed on the nuclear dsDNA stained with DAPI (blue). v = vacuole-like structures.

MCF-7 and NCI-H460 cell morphology was examined under fluorescence microscopy following 24 hours exposure to a single concentration (100 μM) of each drug at which a significant reduction in viable cell number was evident in MTT assays (Figure 2 and Figure 3, respectively). MCF-7 cells exposed to combretastatin A4 were smaller in size, more rounded in shape and notably less well attached to the growth surface when compared to control cells exposed to 1% DMSO; the number of cytoskeletal attachments were also less numerous and the cell margins appeared irregularly shaped. Cell nuclei showed evidence of pyknotic DNA condensation and were generally smaller in size when compared to nuclei of cells in the control group. In thalidomide treated MCF-7 cells both the cytoskeletal and nuclear morphology were largely normal. In OGT 2115 treated MCF-7 cells the cytoskeletal staining showed evidence of swelling and rupture of cells; the integrity of cytoskeletal structure was completely compromised and F-actin appeared fragmented and widely dispersed. Similarly, nuclei displayed characteristic features of dissolution (karyolysis); nuclei were much larger in area and flattened in appearance when compared to control cell nuclei, and there were numerous large vacuole-like regions present that were devoid of the DAPI stain. In cells exposed to tranilast there was no obvious evidence of changes to either cytoskeletal or nuclear morphology and they had an overall appearance similar to that of those in control samples.

In NCI-H460 cells exposed to either combretastatin A4 or OGT 2115 the pattern of cytoskeletal staining was strongly suggestive of necrotic cell death showing signs of cell swelling and rupture; the integrity of cytoskeletal structure was completely compromised and F-actin appeared fragmented and widely dispersed compared to that of control cells treated with 1% DMSO. Similarly, nuclei displayed characteristic features of karyolysis and numerous large vacuole-like regions present that were devoid of DAPI stain. In thalidomide treated cells the cytoskeletal morphology was suggestive of the early stages of apoptosis; the cells were generally smaller and more rounded than control samples and there was some evidence of cytoplasmic blebbing or zeiosis. In addition, both the size of the cell margin and the number of cytoskeletal attachments to the growth surface were visibly reduced. There was no evidence of obvious changes in nuclear morphology. Cells exposed to tranilast showed no obvious evidence of changes to either cytoskeletal or nuclear morphology and had an overall appearance that was similar to that of control cells.

### Combretastatin A4 and thalidomide induced caspase-3 activity

**Figure 4:**
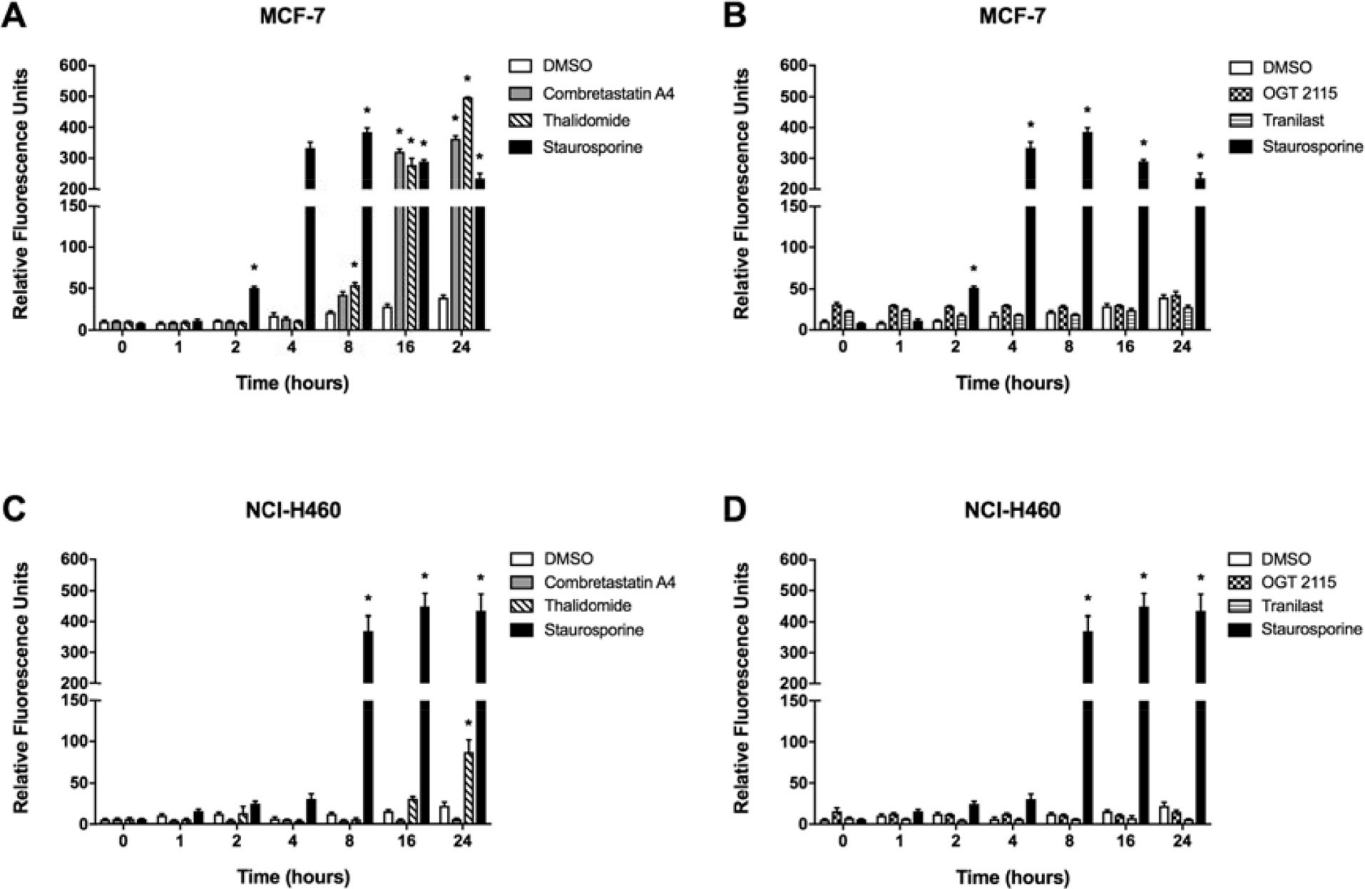
Caspase-3 activity assays Caspase-3 activity in MCF-7 (**A** and **B**) and NCI-H460 cells (**C** and **D**) treated with either 100 μM combretastatin A4, 100 μM thalidomide, 100 μM OGT 2115, 100 μM tranilast or 1 μM staurosporine (as a positive control) at intervals of 0, 1, 2, 4, 8, 16 or 24 hours following treatment. Data are expressed as means ± SEM for three independent experiments (*n* = 3). The differences between control and treatment groups at each time point were determined by two-way ANOVA followed by Dunnett’s multiple comparison test. The asterisk symbol (*) is used to denote statistical significance in the difference between experimental and negative control values (*P* ≤ 0.05).

Caspase-3 activity was measured fluorimetrically over a 24-hour period following treatment of either MCF-7 or NCI-H460 cells with a 100 μM concentration of each anti-angiogenic drug, 1 μM staurosporine (positive control) or 1% DMSO (negative control) (Figure 4). Incubation of MCF-7 cells in the presence of 1 μM staurosporine, a positive control for inducing apoptosis [17], elicited significant caspase-3 activity after 2 hours, which rose to a peak at 8 hours and then gradually declined thereafter; while in the NCI-H460 cell line a marked increase in caspase-3 activity was measured after 8 hours of exposure which remained significantly elevated from 16 hours to 24 hours post-treatment. Treatment of MCF-7 cells with combretastatin A4 induced a significant increase in caspase-3 activity after 16 hours of incubation, which was elevated further at 24 hours. Incubation of cells with thalidomide induced a significant time-dependent increase in caspase-3 activity between 8 and 24 hours. The caspase-3 activity value for cells incubated with thalidomide at 24 hours was greater than that measured for staurosporine at the same time point. Treatment with OGT 2115, tranilast or 1% DMSO did not result in any significant increase in caspase-3 activity throughout the 24-hour time-course. NCI-H460 cells treated with combretastatin A4, OGT 2115 or tranilast did not demonstrate any significant increase in caspase-3 activity throughout the 24-hour time-course. NCI-H460 cells incubated with thalidomide showed a significant increase in caspase-3 activity above basal levels only after 24 hours.

### Combretastatin A4, OGT 2115 and tranilast altered integrated mitochondrial function

**Figure 5:**
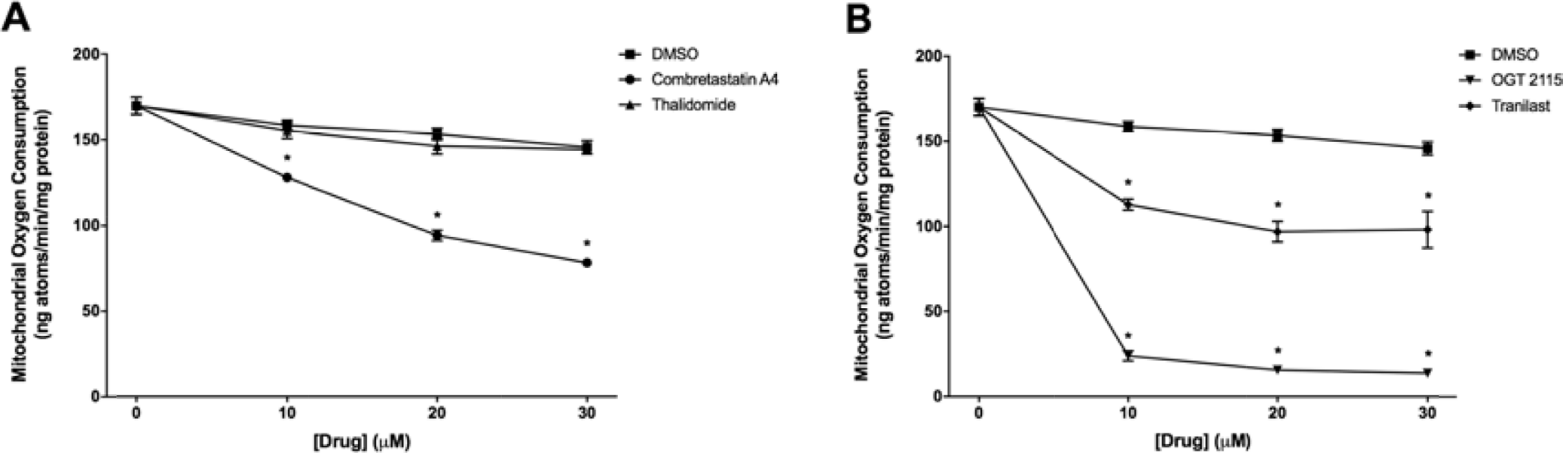
Mitochondrial oxygen consumption measurements Mitochondrial oxygen consumption in state-3 respiration (+ ADP) following sequential addition of either combretastatin A4 (**A**), thalidomide (A) OGT 2115 (**B**) or tranilast (B) to final concentrations of 10 μM, 20 μM and 30 μM. Control samples were exposed to equivalent volumes of DMSO. Data are expressed as means ± SEM for three biologically independent repeat experiments (*n* = 3). The difference between control and treatment groups at each time point was determined by two-way ANOVA followed by Dunnett’s multiple comparison test. The asterisk symbol (*) is used to denote statistical significance in the difference between experimental and negative control values (*P* ≤ 0.05).

**Figure 6:**
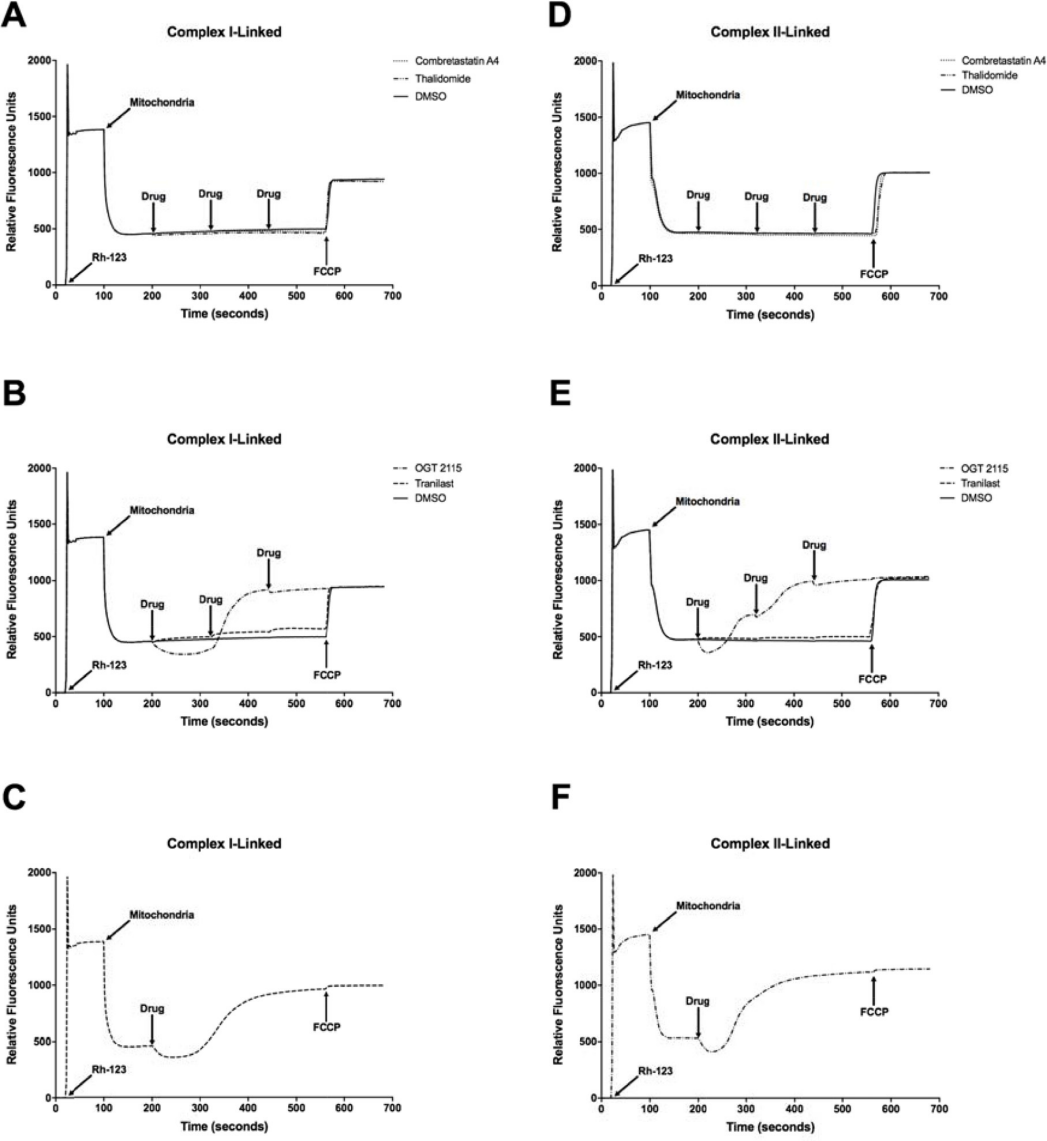
Mitochondrial membrane potential measurements NADH- (**A**–**C**) and FADH_2_-linked (**D**–**F**) mitochondrial membrane potential measurements made using rhodamine-123 (Rh-123) in isolated rat heart mitochondria. On addition of mitochondria to the fluorimeter cuvette, the fluorescence intensity decreased as rhodamine-123 partitioned into the matrices of energised mitochondria. Three sequential additions of each drug or DMSO (vehicle control) were made in each experiment to achieve final concentrations of 10 μM, 20 μM and 30 μM of combretastatin A4, thalidomide, OGT 2115 and tranilast or equivalent concentrations of DMSO (final concentrations of 0.1%, 0.2% and 0.3% (v/v) in solution, respectively). Subsequently, the mitochondrial uncoupling agent FCCP was used to completely depolarise the mitochondria and dissipate any remaining membrane potential. The fluorimetric traces displayed are representative of *n* = 3 independent experiments.

Combretastatin A4, OGT 2115 and tranilast all induced a significant concentration-dependent decrease in mitochondrial oxygen consumption relative to the small change induced by DMSO alone (Figure 5). Thalidomide did not significantly alter the mitochondrial oxygen consumption rate. In addition, no significant change in either complex I-linked or complex II-linked mitochondrial membrane potential (Figure 6) was observed following three sequential 10 μM additions of either combretastatin A4 or thalidomide compared to control measurements made in the presence of equivalent volumes of DMSO. In both cases, addition of the mitochondrial-uncoupling agent FCCP depolarised the mitochondria, completely dissipating the mitochondrial membrane potential and resulting in a large increase in fluorescence that indicated that no membrane potential had been lost following drug exposure. Sequential addition of tranilast at 10 μM incremental concentrations resulted in corresponding small increases in fluorescence intensity, indicating a small concentration-dependent decrease in mitochondrial membrane potential. Addition of OGT 2115 (f.c. 10 μM) initially resulted in a decrease in fluorescence, indicating mitochondrial hyperpolarisation, i.e. an increase in the mitochondrial membrane potential, after which membrane potential appeared to return to the basal level. On further addition of OGT 2115 (f.c. 20 μM) a large and rapid increase in fluorescence was elicited, corresponding to a rapid loss of mitochondrial membrane potential. The final addition of OGT 2115 (f.c. 30 μM) produced minor hyperpolarisation followed by the loss of virtually all remaining membrane potential. Addition of the mitochondrial uncoupling agent FCCP resulted in very little further increase in fluorescence, indicating that virtually all of the membrane potential had previously been dissipated by OGT 2115. The same pattern of hyperpolarisation and subsequent complete depolarisation was achieved when a single addition of OGT 2115 was made to achieve a final concentration of 10 μM.

### All anti-angiogenic compounds tested inhibited mitochondrial complex I activity

**Figure 7:**
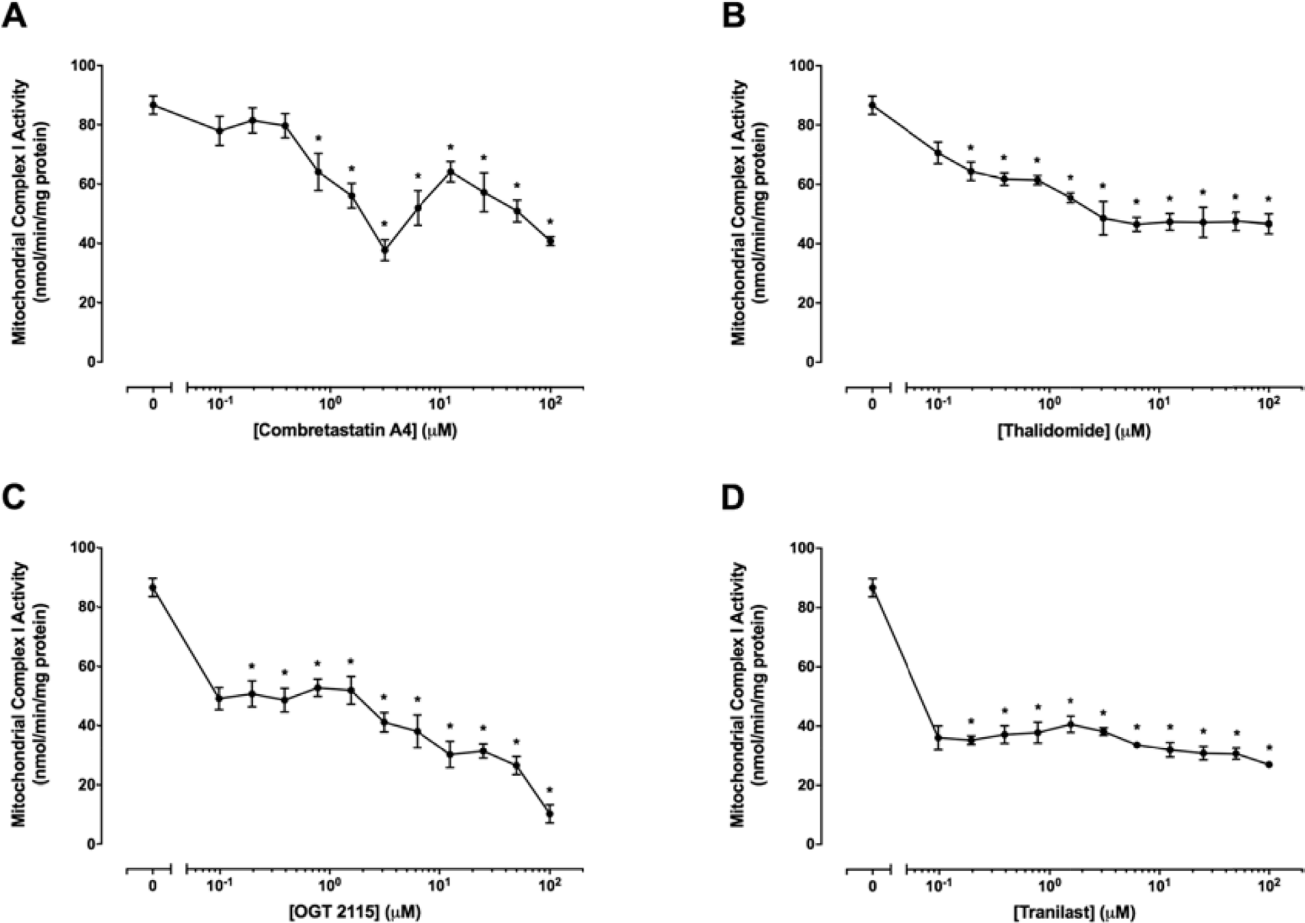
Mitochondrial complex I activity assays The activity of mitochondrial complex I in the presence of a range of concentrations (0.01 μM – 100 μM) of combretastatin A4 (**A**), thalidomide (**B**), OGT 2115 (**C**) or tranilast (**D**) is depicted relative to activity measurements recorded for control samples exposed to 1% DMSO. Data are expressed as means ± SEM for four biologically independent repeat experiments (*n* = 4). The difference between control and treatment groups at each drug concentration was determined by two-way ANOVA followed by Dunnett’s multiple comparison test. The asterisk symbol (*) is used to denote statistical significance in the difference between values recorded for experimental and control samples (*P* ≤ 0.05).

Mitochondrial complex I activity was measured in the presence of a range of concentrations (0.01 μM–100 μM) of combretastatin A4, OGT 2115, thalidomide or tranilast relative to activity measurements recorded for control samples exposed to 1% DMSO (Figure 7). Mitochondrial complex I activity was not significantly changed at concentrations of combretastatin A4 below 0.8 μM, but was significantly reduced, in a tri-phasic manner, at concentrations of 1.6 μM – 100 μM. Significant decreases in mitochondrial complex I activity were measured at all concentrations of OGT 2115 used and the inhibition of mitochondrial complex I activity was increased in a concentration-dependent manner at OGT 2115 concentrations above 0.4 μM. Mitochondrial complex I activity was significantly reduced at all concentrations of thalidomide and tranilast used.

### OGT 2115 inhibited mitochondrial complex II–III activity

**Figure 8:**
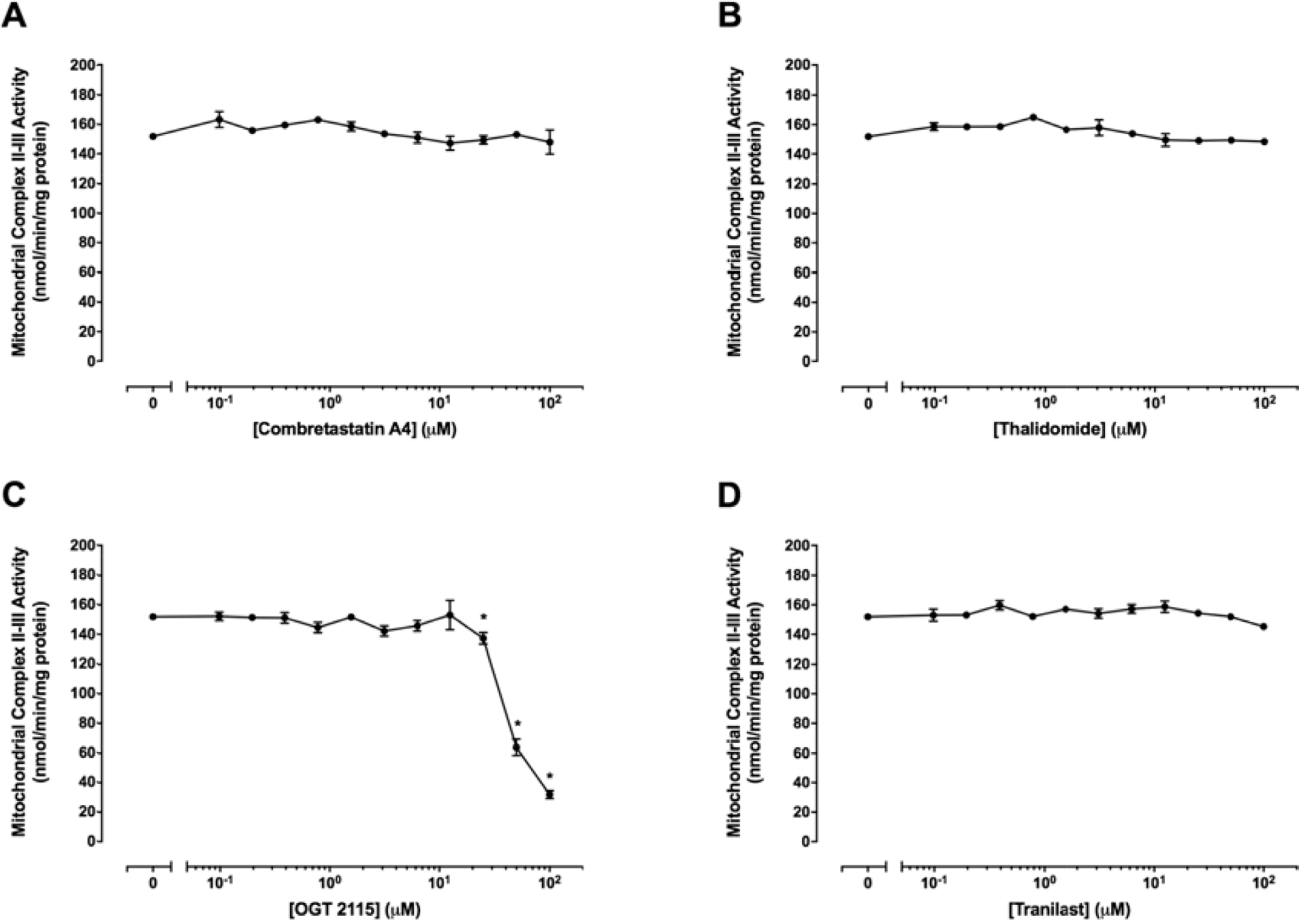
Mitochondrial complex II–III activity assays The activity of mitochondrial complex II–III in the presence of a range of concentrations (0 μM–100 μM) of combretastatin A4 (**A**) thalidomide (**B**) OGT 2115 (**C**) or tranilast (**D**) is depicted relative to activity measurements recorded for control samples exposed to 1% DMSO. Data are expressed as means ± SEM for four independent experiments (*n* = 4). The difference between control and treatment groups at each drug concentration was determined by two-way ANOVA followed by Dunnett’s multiple comparison test. The asterisk symbol (*) is used to denote statistical significance in the difference between values recorded for experimental and control samples (*P* ≤ 0.05).

Figure 8 shows mitochondrial complex II–III activity in the presence of a range of concentrations (0 μM –100 μM) of combretastatin A4, thalidomide, OGT 2115 or tranilast. Combretastatin A4, thalidomide and tranilast did not cause significant alterations in mitochondrial complex II–III activity at any of the concentrations of drugs used. Mitochondrial complex II–III activity was not significantly changed at concentrations of OGT 2115 below 25 μM but was significantly decreased at concentrations of 25 μM–100 μM.

### Combretastatin A4, thalidomide and OGT 2115 altered mitochondrial complex IV activity

**Figure 9:**
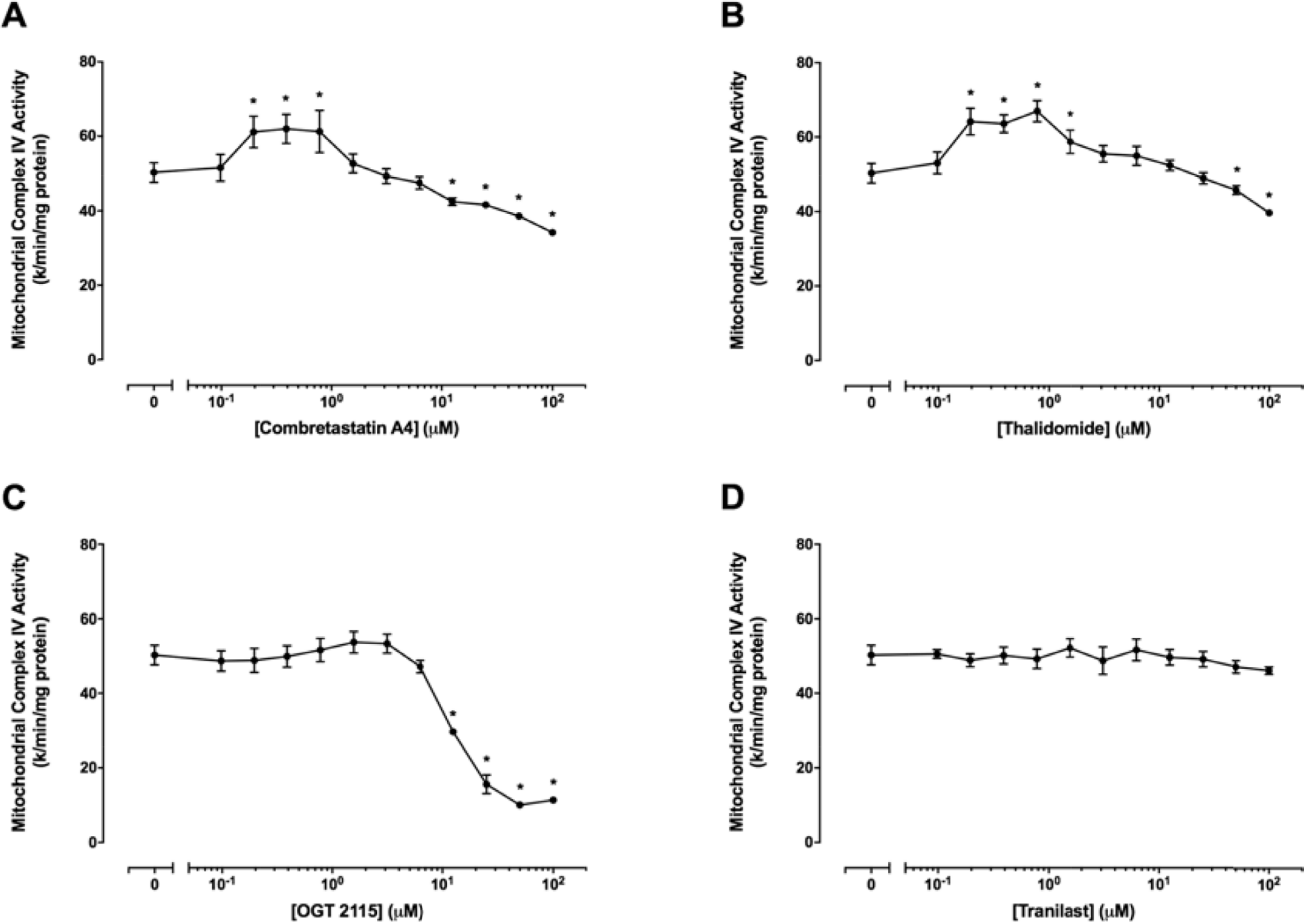
Mitochondrial complex IV activity assays The activity of mitochondrial complex IV in the presence of a range of concentrations (0 μM–100 μM) of combretastatin A4 (**A**) thalidomide (**B**) OGT 2115 (**C**) or tranilast (**D**) is depicted relative to activity measurements recorded for control samples exposed to 1% DMSO. Data are expressed as means ± SEM for four independent experiments (*n* = 4). The difference between control and treatment groups at each drug concentration was determined by two-way ANOVA followed by Dunnett’s multiple comparison test. The asterisk symbol (*) is used to denote statistical significance in the difference between values recorded for experimental and control samples (*P* ≤ 0.05).

Figure 9 shows mitochondrial complex IV activity in the presence of a range of concentrations (0 μM – 100 μM) of combretastatin A4, thalidomide, OGT 2115 or tranilast. Mitochondrial complex IV activity was significantly increased at combretastatin A4 concentrations of 0.01 μM – 0.4 μM but was significantly decreased at concentrations above 12.5 μ M. Similarly, thalidomide concentrations of 0.01 μM – 0.8 μM caused a significant increase in mitochondrial complex IV activity, while concentrations in excess of 50 μM resulted in significantly decreased activity. OGT 2115 caused dose-dependent decreases in mitochondrial complex IV activity at concentrations above 12.5 μM. Tranilast did not cause any alteration to mitochondrial complex IV activity relative to control measurements.

## DISCUSSION

### Combretastatin A4

Combretastatin A4 has been reported to elicit significant cytotoxic effects in numerous cancer cell lines, including both MCF-7 and NCI-H460 lineages [18–20]. Mendez-Callejas *et al.* [21] demonstrated that combretastatin A4 leads “to (mitochondrial) cytochrome c release, alteration in the mitochondrial membrane polarisation, cell cycle arrest at the G_2_/M-phase, and cell death”. Previous studies are consistent with the data shown here, in which both MCF-7 and NCI-H460 cell viability was markedly reduced at concentrations of combretastatin A4 exceeding 1nM. MCF-7 cells treated with combretastatin A4 demonstrated morphological changes which included cell shrinkage, loss of attachment to the growth surface and early stages of nuclear condensation; changes which are typically characteristic of apoptosis [22]. In addition, combretastatin A4 induced significantly increased caspase-3 activity between 8 and 16 hours following exposure to combretastatin A4. When taken together, these data suggest that combretastatin A4 reduced MCF-7 cell viability by induction of apoptosis. NCI-H460 cells treated with combretastatin A4 demonstrated cytoplasmic swelling, loss of normal cytoskeletal structure, evidence of cell membrane rupture, and karyolytic nuclear dissolution; changes which are typically characteristic of necrosis [23]. In addition, combretastatin A4 did not induce any caspase-3 activity in NCI-H460 cells over 24 hours of exposure. When taken together, these data indicate that combretastatin A4 reduced adherent NCI-H460 cell viability by induction of necrotic cell death.

Combretastatin A4 had the same effect on cell death at 1 nM and at 100 μM in both the NCI-460 and MCF-7 cells lines. However, it has been shown that there is a small population in both NCI-460 cells [24] and MCF-7 cells [25] that have features of cancer stem cells, such as increased resistance to drugs. Therefore, these data could indicate that the cells left following treatment with the various drugs are in fact a cancer stem cell sub-population.

While significant decreases in mitochondrial complex I activity were measured in the presence of combretastatin A4, even at relatively low concentrations, there was no evident change in mitochondrial membrane potential under either mitochondrial complex I-linked (NADH-linked) or complex II-linked (FADH_2_-linked) respiration at combretastatin concentrations of 10 μM to 30 μM. These observations might be at least partially explained by the mitochondrial threshold hypothesis, as described by Calabrese *et al.* [26], which proposes that the activity of a particular respiratory chain complex must be decreased to a critical “threshold” level before a significant decrease in integrated mitochondrial function (mitochondrial oxygen consumption, transmembrane potential or ATP synthesis) is able to occur. An additional explanation is one suggested by Abdel-Razaq *et al.* [27] where similar data were obtained during an investigation of the effects of tianeptine, a tricyclic antidepressant, on mitochondrial function. In this study it was suggested that the up-regulation of electron supply to the respiratory chain in the form of reduced flavin adenine dinucleotide (FADH_2_) causes partial or complete bypass of mitochondrial complex I, thus facilitating maintenance of mitochondrial membrane potential regardless of a small decrease in mitochondrial complex I activity.

Combretastatin A4 did not induce any significant reduction in mitochondrial complex II–III activity at any of the concentrations used. This observation was also reflected in the absence of alterations to mitochondrial membrane potential measured in isolated intact mitochondria. Significant concentration-dependent bi-phasic alterations to mitochondrial complex IV activity were observed when isolated mitochondria were treated with increasing concentrations of combretastatin A4. The increase in mitochondrial complex IV activity that occurred between concentrations of 0.01 μM and 0.4 μM may have been due to changes in mitochondrial membrane fluidity; alterations in the fluidity of biological membranes are known to alter the maximum rate of an enzyme-catalysed reactions (V_max_) and the substrate concentration at which these proceed at half-maximal velocity (K_m_) [28]. Furthermore, multi-phasic, drug concentration-dependent changes in mitochondrial complex activity similar to those measured here have previously been reported in relation to the effects of cannabinoid receptor agonists anandamide and Δ-9-tetrahydrocannabinol on the activity of mitochondrial complex I and complex II–III [10]. The decreased complex IV activity induced by combretastatin A4 at concentrations of 12.5 μM and above was likely due to a direct inhibitory effect following stabilisation of the increased fluidity of the membrane that occurred at lower concentrations [10].

Mitochondrial oxygen consumption was reduced by approximately 25–55% in the presence of combretastatin A4 at concentrations of 10 μM to 30 μM. Given that approximately 95% of cellular oxygen consumption occurs as a consequence of the redox reaction between electrons, protons and molecular oxygen at mitochondrial complex IV, this observation would usually provide a strong indication for effective blockade of mitochondrial electron transport [29]. The fact that mitochondrial membrane potential was unchanged is not too surprising, as it is possible to have decreases in mitochondrial oxygen consumption in the absence of changes in mitochondrial membrane potential. Mitochondrial oxygen consumption is a much more sensitive indicator of oxidative phosphorylation and the levels of inhibitors required to effect a change are much lower than those required to cause an immediate change in mitochondrial membrane potential [30]**.**

An alternative explanation is that reactive derivatives of combretastatin A4 were generated within the electrode incubation chamber and the oxidation of these compounds was able to lead to the direct consumption of oxygen via a chemical reaction. In support of this, it has been reported that two ortho-quinone derivatives of combretastatin A1 (a structural analogue of combretastatin A4) are generated in the presence of glutathione by the oxidative action of peroxidase enzymes. These compounds were shown to cause significant reductions in oxygen tension during polarographic measurements and this effect was significantly increased in the presence of glutathione [31]. Mitochondria are known to contain a number of peroxidase enzymes (including glutathione peroxidase and cytochrome *c* peroxidase) and glutathione levels at concentrations comparable to those of the cytosol (approximately 5 mM) [32–34]. Therefore, it is possible that reactive combretastatin A4 derivatives could have been generated in solution during the polarographic measurement of mitochondrial oxygen consumption, and that this subsequently led to the observed reduction in oxygen levels in the absence of altered mitochondrial membrane potential.

### Thalidomide

Previous studies have demonstrated a mitochondrial effect of thalidomide and its analogues on the Jurkat T cell line, used for the study of acute T cell lymphoma [35]. Our data supports this, as thalidomide was able to reduce MCF-7 cell viability and induced a significant increase in caspase-3 activity at around 8 hours post-exposure. However, unlike combretastatin A4, morphological changes indicative of apoptosis were not evident after 24 hours exposure to 100 μM thalidomide. Furthermore, thalidomide was only able to decrease NCI-H460 cell viability at a concentration of 100 μM. It has previously been reported that thalidomide was able to cause an approximate 50% reduction in NCI-H460 cell viability at a concentration of 3.87 μM [36]; a much lower concentration than that seen to reduce cell viability in this present study. However, the cytotoxicity assays previously reported were undertaken over a much longer duration [36]. These contrasting findings suggest that the cytotoxic effects of thalidomide *in vitro* are both concentration-dependent and time-dependent. Thalidomide is regarded as a pro-apoptotic agent, and this cell death modality has been reported previously in a NCI-H460 cancer cell model [37]. The data from the caspase-3 activity assays indicate that apoptosis was the mechanism of cell death in the presence of thalidomide, which led to reductions in MTT staining measured in the NCI-H460 cells. Furthermore, in the NCI-H460 cells cultured in the presence of thalidomide, our data showed morphological changes characteristic of apoptosis.

Thalidomide elicited a concentration-dependent decrease in mitochondrial complex I activity at concentrations of 0.1 μM to 6.25 μM and maintained decreased activity at around 55% of control values between 6.25 μM and 100 μM. While significant decreases in mitochondrial complex I activity were measured at thalidomide concentrations of 10 μM to 30 μM, these concentrations did not produce a significant decrease in mitochondrial oxygen consumption or transmembrane potential. The mitochondrial threshold hypothesis and the effective bypass of mitochondrial complex I, as proposed for combretastatin A4, could also explain these observations. Bi-phasic modulation of complex IV activity by thalidomide was observed, similar to that which was observed in the case of combretastatin A4. However, in the case of thalidomide no reduction in mitochondrial complex II–III activity was present.

### Tranilast

Tranilast concentrations of 10 μM to 30 μM resulted in small concentration-dependent reductions in complex II-linked mitochondrial membrane potential in isolated rat heart mitochondria. These reductions in mitochondrial membrane potential may have been the result of inhibition of respiratory chain components or non-respiratory chain mitochondrial enzymes, such as those of the tricarboxylic acid cycle (TCA cycle). In addition, the rotenone-induced inhibition of complex I necessary for measurement of transmembrane potential under FADH_2_-linked respiration may have potentiated this effect; the inhibition of complex I activity is known to lead to an increased intra-mitochondrial NADH/NAD^**+**^ ratio [38, 39] and the subsequent feedback inhibition of the TCA cycle would lead to a reduction in mitochondrial FADH_2_ levels, thus making the effects of any further inhibition of the TCA cycle more potent.

Tranilast was able to induce significant inhibition of mitochondrial complex I activity at all concentrations used. This decrease in activity was maintained at around 40–45% of control values at concentrations between 0.098 μM and 3.125 μM, after which a concentration-dependent reduction in complex I activity was evident. At 100 μM tranilast, the activity of complex I was reduced to approximately 30% of control values. In addition, tranilast concentrations of 10 μM to 30 μM resulted in a 34–44% decrease in mitochondrial oxygen consumption, and concentration-dependent reductions in mitochondrial transmembrane potential in the presence of complex I-linked respiration. All of these data suggests that tranilast is a specific mitochondrial complex I inhibitor as there were no significant reductions in mitochondrial complex II–III or mitochondrial complex IV activity measured in the presence of tranilast.

In the MCF-7 cell model, there was no evidence of a significant decrease in cell viability at tranilast concentrations of 1 nM to 10 μM. However, viable cell mass was significantly reduced, to around 75% of control values, at a tranilast concentration of 100 μM. These data are consistent with the study of Darakhshan and Ghanbari [12], who showed that 100 μM tranilast was able to effect a comparable 20–40% reduction in MCF-7 cell viability after 48 hours exposure. They also demonstrated clear evidence of apoptosis as the predominant cell death mechanisms that led to the observed reduced cell viability. In contrast, the data presented here did not indicate any obvious morphological changes under fluorescence microscopy, nor was there any evidence of tranilast-induced changes in caspase-3 activity over time.

While caspase-dependent apoptotic cell death in the presence of tranilast has been demonstrated in other human breast cancer cell lines [40], it still remains unclear as to how tranilast-induced apoptosis is mediated in the MCF-7 cell line. However, cathepsin-dependent (caspase-independent) apoptosis has been demonstrated previously [41]. Hence, the low caspase-3 activity in conjunction with significantly reduced viable cell mass may indicate caspase-independent apoptosis being induced by tranilast in the MCF-7 cell model [41]. An alternative explanation is that the small inhibition of complex I and resultant changes in integrated mitochondrial functions demonstrated at high tranilast concentrations are enough to significantly decrease ATP production such that cell proliferation is slowed and the number of viable cells present in treated samples is reduced compared to controls in the absence of cytotoxic effects.

In contrast to the findings in the MCF-7 cell line, tranilast did not elicit any reduction in cell viability in the NCI-H460 cell line. Cell morphology appeared unaltered after 24 hours in the presence of 100 μM tranilast, and caspase-3 activity was not increased over the same period of time at this concentration. At present there is a lack of other scientific literature data with which to compare these observations. However, NCI-H460 cells are known to show metabolic adaptations for significantly increased glycolytic energy metabolism, and such a glycolytic phenotype is associated with resistance to apoptosis [42]. Therefore, it seems likely that NCI-H460 cells were able to circumvent any reduction in mitochondrial oxidative phosphorylation induced by tranilast by increasing glycolytic ATP synthesis to meet cellular energy requirements, thus facilitating cell survival.

### OGT 2115

When exposed to 10 μM OGT 2115, the oxygen consumption rate of isolated rat heart mitochondria was significantly inhibited. Further addition of OGT 2115 caused very little change to the already largely decreased oxygen consumption rate. A single 10 μM dose of OGT 2115 also caused a rapid mitochondrial hyperpolarisation that was followed by a large, rapid depolarisation and an almost complete collapse of mitochondrial membrane potential. As with oxygen consumption measurements, the pattern of change in mitochondrial membrane potential occurred independent of increased OGT 2115 concentration. The initial hyperpolarisation of the mitochondrial membrane observed following addition of OGT 2115 to isolated mitochondria suggests inhibition of the function of the mitochondrial ATP synthase complex (mitochondrial complex V), similar to that known to be elicited by the classical complex V inhibitor oligomycin [43]; protons are blocked from entering the mitochondrial matrix and thus are sequestered outside of the inner mitochondrial membrane causing a resultant hyperpolarisation and also diminished utilisation of oxygen at mitochondrial complex IV. Inhibition of complex V leads to a hyperpolarisation of the mitochondrial membrane potential [44], followed by its collapse due to the electrostatic breakdown of the potential (effectively electrical “arcing”) as the charge density across the mitochondria inner membrane becomes too large. This is followed often by the physical breakdown of the mitochondrial inner membrane, and its lysis, leading to a complete collapse of the inner mitochondrial membrane potential [30, 45].

OGT 2115 was shown to induce a concentration-dependent reduction in the activity of mitochondrial complex I, complex II–III and complex IV. Inhibition of the mitochondrial respiratory chain at complex I and/or complex III in particular is known to increase the mitochondrial production of reactive oxygen species (ROS) [46, 47]. The rapid release of ROS that would likely result from the actions of OGT 2115 is likely to overwhelm mitochondrial and cellular reactive oxygen species detoxification modalities such as superoxide dismutase, glutathione peroxidase, catalase, glutathione, vitamin C, vitamin E and other free radical scavenging systems, resulting ultimately in significant mitochondrial and cellular damage and a direct adverse effect on cellular integrity.

The data obtained from both MCF-7 and NCI-H460 cell models supports the suggested mitochondrial mechanisms of OGT 2115 action. The significant reduction in cell viability at OGT 2115 concentrations greater than 0.1 μM is suggestive of an agent that has a significant effect on cellular physiology. The changes to the morphology of the remaining adherent MCF-7 and NCI-H460 observed under fluorescence microscopy were characteristic of necrosis and the complete absence of changes in caspase-3 activity over the time frame of the experiments strengthens the argument for necrosis being the predominant cell death modality leading to the recorded reduction in viable cancer cell mass in the presence of OGT 2115.

OGT 2115 has been shown to be an inhibitor of the enzyme heparanase [14, 48]. However, OGT 2115 has been little studied, and It is clear from the emerging scientific literature that the specific molecular targets and actions of many drugs including the four drugs we specifically chose are not completely understood and that although, for instance, OGT 2115 is a heparanase inhibitor, that may in fact not be its major mechanism of action in cancer cells either *in vitro* or *in vivo*. Many drugs have been shown to have multiple targets [49], and our focus has been on drugs that could potentially affect mitochondrial function and cause selective cancer cell death, whilst sparing normal cells.

The data presented here strongly supports the hypothesis that the anti-angiogenic drugs combretastatin A4, thalidomide, OGT 2115 and tranilast all have mitochondrial targets in addition to their documented actions as anti-angiogenic agents. Our data show decreases in oxygen consumption in the presence of combretastatin A4 and tranilast without significant changes in mitochondrial membrane potential. This is in keeping with what is already known concerning control of oxidative phosphorylation, as large changes in oxygen consumption may be associated with relatively small changes in mitochondrial membrane potential due to the relationship between the fluxes through the three essential components responsible for the control of oxidative phosphorylation, those of substrate oxidation, ATP turnover and proton leak [30]. Mitochondrial membrane potential and mitochondrial oxygen consumption are not inextricably linked, as the factors that control one do not always control the other. It is possible to maintain a steady mitochondrial membrane potential in the presence of decreased electron transport, as other factors besides passage of electrons down the respiratory chain control mitochondrial membrane potential. These include electron slip and leak associated with uncoupling proteins, as well as substrate and ion transport by the malate aspartate shuttle, the adenine nucleotide transporter and the calcium uniporter [50], all of which can affect the mitochondrial membrane potential [26, 30]. In the case of the anti-angiogenic compounds combretastatin A4 and tranilast, it is possible that these compounds are inhibitors of mitochondrial uncoupling proteins, able to inhibit proton leak, stabilising mitochondrial membrane potential, whilst also decreasing mitochondrial complex I activity.

Our data indicate that combretastatin A4, thalidomide, OGT 2115 and tranilast, all anti-angiogenic drugs with diverse molecular structures and reported biological actions, are able to directly affect cancer cell viability and also have effects on mitochondrial function. While the ability to modulate mitochondrial physiology does not always appear to translate to direct anti-cancer action in a two-dimensional MCF-7 and NCI-H460 cell models *in vitro*, OGT 2115 and tranilast show clear evidence of having direct effects on mitochondrial activity that are able to cause a direct reduction in cancer cell viability. Tranilast is a mitochondrial complex I inhibitor and OGT 2115 has significant effects on the integrated function of isolated mitochondria that translate into potent cytotoxic action; potentially by causing mitochondrial dysfunction through increased ROS production and/or causing formation of the mitochondrial permeability transition pore [26, 50]. The differences in the observed effects of these compounds highlight the diverse array of potential mechanisms by which mitochondrial physiology may be modulated to achieve anti-cancer action. Furthermore, the observation that anti-angiogenic drugs, originally thought to act via several extra-mitochondrial pathways, do in fact possess significant ability to modulate mitochondrial function clearly adds significant credibility to the hypothesis that a considerable proportion of the action of some anti-angiogenic drugs might be at mitochondrial sites. When taken together, these observations strengthen the case for the future investigation and development of anti-cancer therapies that specifically target cancer cell mitochondria for both inhibition of angiogenesis and stimulation of cell death.

There appears to be no scientific literature available (apart from our control data), on the concentration dependent properties of DMSO on intact isolated mitochondrial. However, previous studies on cultured astrocytes have shown that exposure of cultured astrocytes to 1% DMSO for 24 hours decreased cell viability and caused mitochondrial swelling, decreased mitochondrial membrane potential increased reactive oxygen species production, and subsequent cytochrome c release and caspase-3 activation [51]. These data support our previous assertions that drugs and their solvents can indeed modulate mitochondrial membrane fluidity [9, 10]. Further work on DMSO itself has demonstrated that in phospholipid membrane models, DMSO is observed to exhibit three distinct modes of action, each over a different concentration range. At increasing concentrations, DMSO induces membrane thinning and increases fluidity of the membrane’s hydrophobic core, the induction of transient water pores, the desorption of individual lipid molecules the membrane, and finally by disintegration of the bilayer structure [52].

Little is known of tissue drug concentrations in man during cancer chemotherapy, as this would entail potentially ethically unacceptable serial biopsies of tissue. However, tissue drug concentrations are likely to vary very much depending on differences in tissue and cellular (cancer versus normal cell) metabolism, for example liver and other tissues having a variety of drug metabolism enzyme systems including cytochrome P450’s. We have specifically chosen to use a concentration of 100 μM in some of the studies detailed here and this is because we feel that tissue (as opposed to plasma) concentrations of drugs in man are likely to be much higher than previously thought. This has been demonstrated by Weigman *et al.* [53], who showed that the concentration of clomipramine and its active metabolite desmethylclomipramine reaches almost 100 μM *in vivo*, in rat brain tissue, 4 hours after the last oral dose of clomipramine.

In conclusion, we feel that the data presented in this manuscript strongly supports the increasing evidence that many drugs have complex molecular mechanisms of action and more than one specific receptor/target, some of which are able to modulate cell metabolism at the mitochondrial level and bring about cancer cell death.

## MATERIALS AND METHODS

Eagle’s Minimum Essential Medium (EMEM) basal cell culture medium, RPMI-1640 basal cell culture medium and foetal bovine serum (FBS) were purchased from Life Technologies Ltd. (Paisley, U.K.). All chemical reagents used as described in subsequent sections were of the highest quality available and were sourced from Sigma-Aldrich Company Ltd. (Dorset, U.K.). Combretastatin A4, thalidomide and tranilast were sourced from Abcam plc. (Cambridge, U.K.). OGT 2115 was sourced from Tocris Bioscience (Bristol, U.K.). All anti-angiogenic drug compounds were dissolved in dimethyl sulphoxide (DMSO) to produce stock solutions of 10mM. Stock solutions were diluted in either cell culture medium or the appropriate assay buffer in order to achieve the desired final concentrations used during each experimental procedure.

### Cell culture

MCF-7 and NCI-H460 cells were obtained as fully authenticated cryogenically frozen cultures from the American Type Culture Collection (ATCC) (Manassas, VA, U.S.A.). MCF-7 cells were maintained *in vitro* as adherent monolayer cultures in a complete cell culture medium comprised of EMEM supplemented with 10% (v/v) FBS while NCI-H460 cells were maintained as adherent monolayer cultures in a complete medium comprised of RPMI-1640 supplemented with 10% (v/v) FBS. Cell culture medium was maintained at physiological pH and temperature by equilibration with a mixture of 5% CO_2_ and 95% air at 37°C inside a humidified water-jacketed incubator. Nutrient-depleted culture medium was exchanged for an equal volume of fresh complete medium at 48-hour intervals. Cells were sub-cultured or harvested for use at 70–80 % confluency by incubation at 37°C with 0.25% trypsin-EDTA solution for 5 minutes.

### MTT assay

The cytotoxic effect of each anti-angiogenic drug was determined using an MTT assay. Cells were seeded into 96-well cluster plates at a density of 2.0 × 10^3^ cells per well in a 200 µl volume of complete medium and placed into incubation at 37°C overnight. Spent culture medium was removed following overnight incubation and replaced with an equal volume of complete medium containing a range of concentrations (1 nM–100 µM) of anti-angiogenic drug. Growth controls (cells with culture medium), vehicle controls (cells with culture medium + 1% DMSO) and blank wells (culture medium only) were included on each plate. Plates were then replaced in the incubator for 72 hours. After a 72-hour incubation period, the MTT assay procedure was undertaken according to the method described previously [8], except that incubation time in the presence of MTT solution was adjusted to 3 hours.

### Fluorescence microscopy

Cells were grown to 70–80% confluency on round glass cover slips (1.5 mm thickness, 12 mm diameter) pre-coated with 150–300 kDa poly-L-lysine in flat-bottomed 24-well cluster plates. The nutrient-depleted medium was removed from each well and replaced with an equal volume of complete medium containing 100 µM of anti-angiogenic drug or 1% DMSO in complete medium (negative control). A further 24-hour incubation period was then undertaken, after which the drug-containing medium was removed from all wells and the adherent cell layer washed three times with phosphate buffered saline (PBS) prior to fixation and staining. Cells were fixed for 15 minutes using 3.7% methanol-free formaldehyde in PBS and then subsequently washed three times in PBS. Cells were permeabilised using 0.1% Triton X-100 in PBS for 5 minutes, followed by three more washes in PBS. Cytoskeletal filamentous actin (F-actin) and double-stranded DNA (dsDNA) were stained using Alexa Fluor^®^ 488-phalloidin and 4′,6-diamidino-2-phenylindole (DAPI) (both sourced from Life Technologies Ltd.) according to the supplier’s instructions. Samples were air dried and mounted onto glass microscope slides in a minimal volume of ProLong^®^ Gold anti-fade mounting medium (Life Technologies Ltd.) according to the supplier’s instructions. All samples were imaged at X1000 magnification using a Nikon Eclipse E-800 upright microscope fitted with a Nikon DS-Fi1 high-definition colour camera head and a Nikon DS-L2 camera control unit (Nikon U.K. Ltd., Kingston upon Thames, U.K.).

### Measurement of Caspase-3 activity

Caspase-3 activity was measured directly by its ability to enzymatically cleave the fluorogenic substrate acetyl-Asp-Glu-Val-Asp-aminomethylcoumarin (Ac-DEVD-AMC), as described previously [8, 27]. Cells were seeded into 60 mm polystyrene tissue culture dishes, supplemented with 5ml of complete culture medium and grown to 70–80% confluency. Once the desired level of coverage was reached, the medium within the dishes was exchanged for an equal volume of complete medium containing either 100 µM of each anti-angiogenic drug, 1% DMSO (negative control) or 1 µM staurosporine, used as a positive control for induction of apoptosis [17]. Dishes were then placed into incubation for time intervals of 0, 1, 2, 4, 8, 16 and 24 hours. At each time-point, dishes were removed from incubation, placed directly on ice and cells harvested using a plastic cell scraper. Both medium and scraped cells were transferred to pre-cooled centrifuge tubes and centrifugation undertaken for 10 minutes at 200 × *g* and 4°C. The resultant supernatant was discarded and each pellet carefully washed twice with ice cold PBS. Cell lysis was achieved by incubation for 30 minutes in ice-cold lysis buffer (100 mM 4-(2-hydroxyethyl)-1-piperazineethanesulfonic acid (HEPES), 100mM sodium chloride, 1 mM EDTA, 0.1% 3-[(3-cholamidopropyl)dimethylammonio]-1-propanesulphonate (CHAPS) and 10% sucrose dissolved in ultrapure (18.2MΩ-cm) distilled water and adjusted to pH 7.4 at 4°C) with continuous agitation. In order to ensure all samples remained chemically reduced, dithiothreitol (DTT) was added to a final concentration of 10 mM on the day of use. Following lysis, samples were subjected to centrifugation at 10,000 × *g* for 10 minutes at 4°C in order to produce a cytosolic fraction (supernatant), which was then carefully removed and stored at −80°C until required for measurement caspase-3 activity. Cytosolic extracts were added to 96-well black polystyrene clear-bottomed fluorescence assay plates. The pan-caspase inhibitor Z-Val-Ala-Asp-fluoromethylketone (Z-VAD-FMK) was added to half of the samples (f.c. 10 µM) and incubation undertaken for 30 minutes on a heated plate shaker maintained at 37°C. Subsequently, Ac-DEVD-AMC was added to all wells (f.c. 20 µM) and further incubation undertaken in complete darkness for 1 hour. Final reaction volume in all wells was 300 µl. The caspase-dependent production of fluorescent 7-amino-4-methylcoumarin (AMC) from the parent AC-DEVD-AMC molecule was measured by the fluorescence intensity at an excitation wavelength of 350 nm and an emission wavelength of 450 nm using a Tecan Infinite 200 Pro Series multimode microplate reader (Tecan UK Ltd., Reading U.K.).

### Isolation of rat heart mitochondria

Rat heart mitochondria were prepared from the hearts of 250 g adult Lister rats using homogenisation and differential velocity centrifugation as previously described [50].

### Measurement of mitochondrial oxygen consumption

Mitochondrial oxygen consumption was measured polarographically using a Clarke-type oxygen electrode (Rank Brothers Ltd., Cambridge, U.K.) in respiration state 3 (+ ADP) at 37°C with 10 mM malate + 10 mM glutamate as substrates, as described previously [54]. 0.25 mg of mitochondrial protein was used in all assays. In separate assays, combretastatin A4, thalidomide, OGT 2115 and tranilast were added to the mitochondrial suspension in the oxygen electrode chamber to achieve final concentrations of 10 µM, 20 µM or 30 µM and incubated for 5 minutes before the addition of ADP.

### Measurement of mitochondrial membrane potential

Mitochondrial membrane potential measurements were made fluorimetrically using 0.2 µM rhodamine-123 at 37°C. Complex I (NADH)-linked changes in mitochondrial membrane potential were measured using 10 mM malate and 10 mM glutamate as substrates while complex II (FADH_2_)-linked changes in mitochondrial membrane potential were made in the presence of 1 µM rotenone using 10 mM succinate as substrate. Both assay procedures were undertaken according to the methods outlined previously [27]. 0.25 mg of mitochondrial protein was used in all assays. The mitochondrial uncoupling protonophore FCCP was used (f.c. 10 µM) in order to completely dissipate any transmembrane potential remaining following the addition of anti-angiogenic agents.

### Measurement of mitochondrial complex I, complex II–III, complex IV activities

Freeze-thawed rat heart mitochondria were re-suspended in a respiration buffer solution (100 mM KCl, 75 mM mannitol, 25mM sucrose, 10 mM Tris, 0.1 mM EDTA, and 10mM phosphate-Tris in ultrapure distilled water, adjusted to pH 7.4 at 37°C) and aliquoted as 240 µl volumes into 96-well cluster plates at 37°C for 15 minutes to thermostabilise. Drugs (or vehicle) were then added in a volume of 10 µl to give a range of concentrations from 0.01 µM to 100 µM and plates were incubated for a further 5 minutes at 37°C to allow for drug binding. Samples were then pipetted off from the drug-mitochondria suspensions and added to separate assay plates containing the components needed for the assay of mitochondrial complex I, complex II–III or complex IV activity. 0.25 mg of mitochondrial protein was used in all assays. The specific activity of mitochondrial complex I (NADH-ubiquinone oxidoreductase) [EC 1.6.5.3] was measured spectrophotometrically as the rotenone sensitive rate of NADH oxidation at 340 nm and 37°C. The activity of mitochondrial complex II–III [EC 1.5.3.1] was measured spectrophotometrically as the antimycin A sensitive rate of cytochrome *c* reduction at 550 nm and 37°C. The activity of mitochondrial complex IV (cytochrome c oxidase) [EC 1.9.3.1] was measured spectrophotometrically as the rate of cytochrome *c* oxidation at 550 nm and 37°C. In all cases, the methods undertaken were as previously described [8].

### Protein assay

Cytosolic and mitochondrial protein concentrations were determined, using a modified microplate version of the Lowry protein assay described previously [8]. Bovine serum albumin (0–200 µg/ml) was used as a concentration standard.

### Statistical analyses

All data displayed are the result of at least *n* = 3 independent experiments. Data are expressed as means ± the standard error of the mean. Statistical analyses were performed using GraphPad Prism 6 (GraphPad Software, Inc., La Jolla, U.S.A.). The statistical test undertaken for each experimental data set is indicated in the respective figure legend. Statistical significance was attributed when a *P*-value of ≤ 0.05 was obtained.
